# Spinal extradural angiolipoma manifested after normal vaginal delivery

**DOI:** 10.1186/s13104-016-1944-3

**Published:** 2016-02-29

**Authors:** Zakaria. I. Mohammed, Moayad. M. Z. Ahmed

**Affiliations:** Omdurman Military Hospital, P.O. Box 2613, 11111 Omdurman, Sudan; Aljerief West 1st block plot number 398, P.O. Box 10995, Khartoum, Sudan

**Keywords:** Spinal angiolipoma, Magnetic resonance imaging, Spinal extradural neoplasm, Spinal cord compression, Vaginal delivery

## Abstract

**Background:**

Extradural spinal angiolipomas are extremely rare benign neoplasms made up of mature lipocytes with abnormal blood vessels. Spinal angiolipomas represent only 0.14–1.2 % of all spinal axis tumours.

**Case presentation:**

A case of thoracic spinal extradural angiolipoma producing acute spinal cord compression in a 35-year old housewife is presented. Patient presented with sudden onset of lower limbs paralysis and urinary incontinence for 1 month after vaginal delivery. Patient was diagnosed as dorsal spine angiolipoma which was treated surgically with excellent outcome.

**Conclusions:**

Spinal angiolipomas are rare tumours but it is mandatory to include it in the differential diagnosis of the spinal extradural space occupying lesions. Pregnancy and vaginal delivery may suddenly exacerbate the condition. The best investigation to choose to diagnose these lesions is definitely magnetic resonance imaging of the spine. The aim of treatment of extradural angiolipomas of the spine is to resect the tumour in Toto surgically with no need of adjuvant therapy, surgery alone can lead to excellent outcome.

## Background

Epidural angiolipomas of the spine are a benign neoplasm composed of mature fat tissues and abnormal vascular architecture, predominantly in middle-aged, female, fat and pregnant patient’s situated mainly in the mid-thoracic region unlike other extradural spinal lipomas. There are only 142 cases with spinal extradural angiolipoma reported since 1890–2013. They represent about 0.14–1.2 % of all axial spine tumours and 2–3 % of spinal epidural tumours [1]. Authors report a rare case of epidural spinal angiolipoma in young female patient presented with complete paraplegia following vaginal child birth which showed a typical appearance of lipoma on magnetic resonance imaging (MRI). The pathology, clinical presentation, diagnostic evaluation and methods pre and post operatively, and treatment of spinal extradural angiolipoma were reviewed.

## Case presentation

35 years old female presented to the casualty with bilateral lower limb paralysis for 1 month following child birth, the condition occurred suddenly, it was associated with back pain and complete loss of sphintric control. On examination patient looks generally well, with normal vital signs readings, GCS 15/15, intact cranial nerves, normal power, tone and reflexes in upper limbs with intact sensation to all modalities, but lower limb showed power grade zero, hypertonia, hyper-reflexia grade 4 and positive Babinski’s sign. Sensory modalities were examined and revealed sensory impairment up to the level of umbilicus (loss of vibration and position sense), and there was mild mid-dorsal tenderness.

**Fig. 1:**
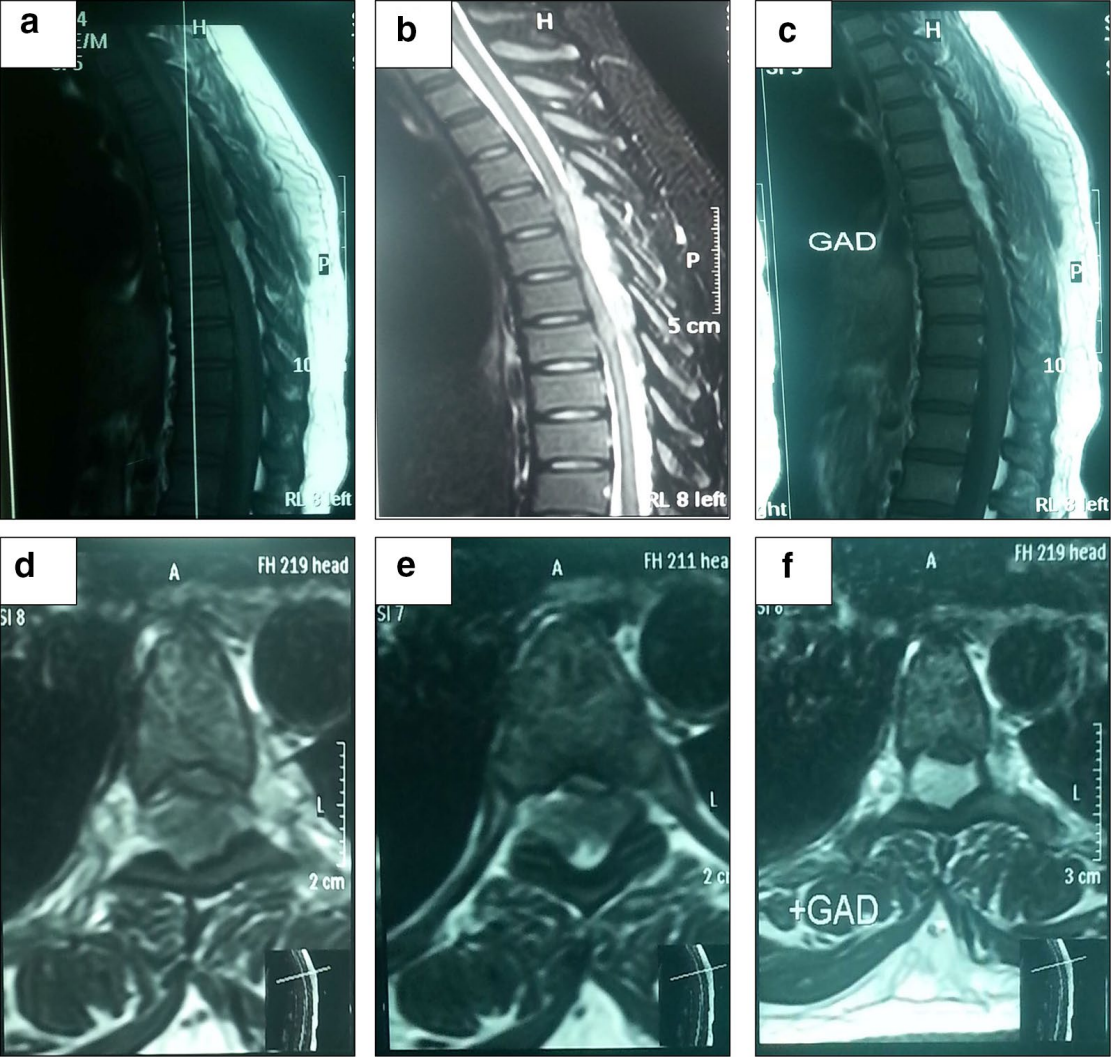
**a** Non-contrast T1-weighted sagittal MRI of the mid thoracic spine showing a very inhomogeneous mass, extending from D5 to D8. The tumor shows a large component hypointense to fat. **b** T2-weighted sagittal MRI showing the mass as hyperintense relative to spinal cord and nearly isointense with normal fat and myelopathic changes in the cord. **c** Post-contrast T1-weighted sagittal MRI showing diffuse, slightly inhomogeneous enhancement of the mass. Non contrast T1-weighted axial MRI (**d**) T2-weighted axial MRI (**e**) and post-contrast T1-weighted axial MRI (**f**) the mass along the posterior epidural spinal canal of the dorasl spine, compressing and displacing the spinal cord anteriorly

**Fig. 2:**
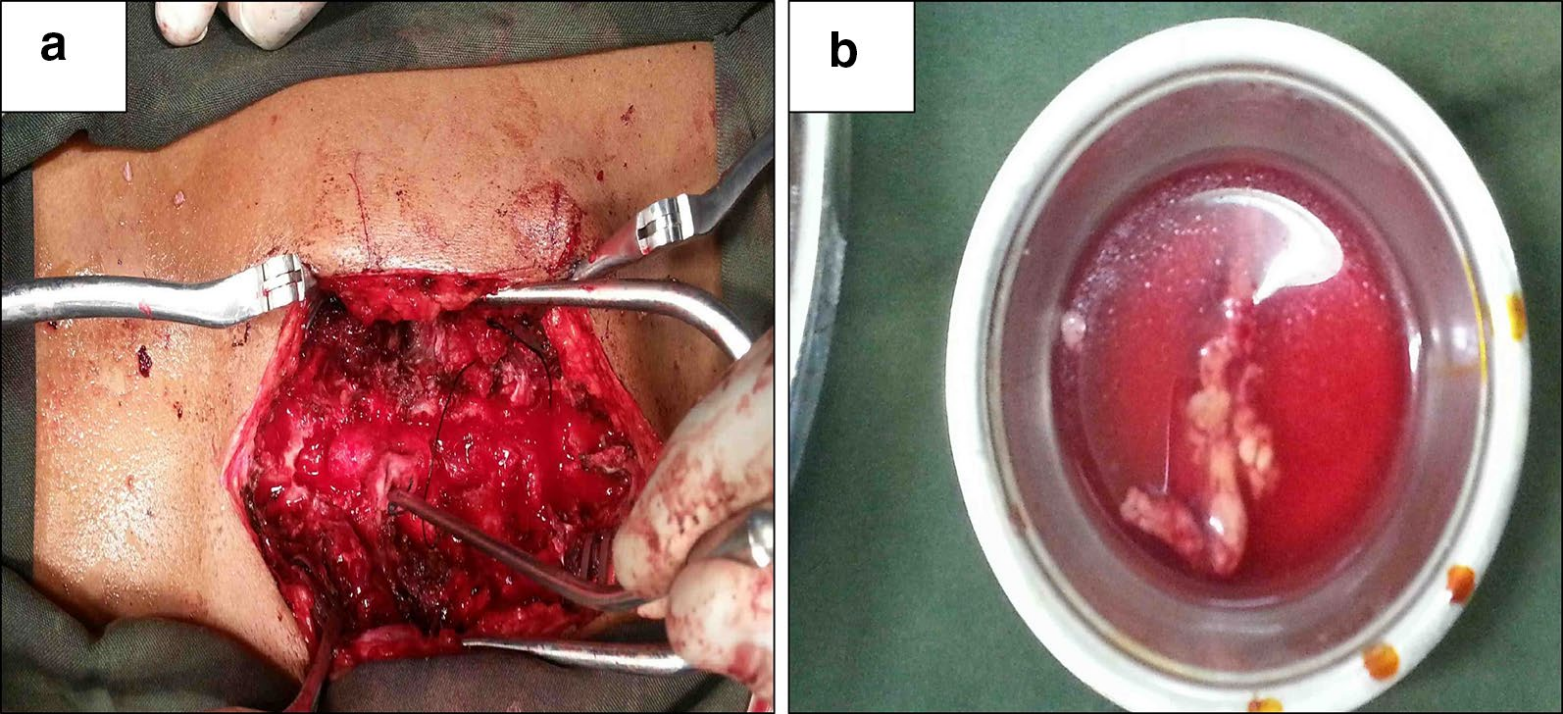
The specimen demonstrated an elongated, *dark-red*, encapsulated mass measuring 9 mm in length 10 mm in width **a** intraoperative image of the tumour before total resection **b** specimen after tumour total removal

Patient haematological parameters were investigated and revealed normal readings. Dorsolumber MRI was done (Fig. 1) showed T1 weighted extradural hyperintense lesion from D5 to D8 compressing the spinal cord from posterior, T2 weighted images showed the lesion which is hyperintense in relation to spinal cord and almost isointense with normal fat signals and cord myelopathic changes and contrasted MRI images showed prevalent, a little inhomogeneous contrast uptake by the lesion. Surgical intervention was done through posterior approach dorsal decompressive laminectomies and total resection of the lesion with no intraoperative significant events was achieved, and specimen was sent for histopathology (Fig. 2).

## Results

**Fig. 3:**
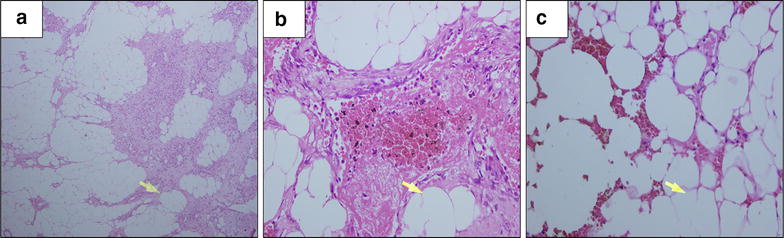
Microscopically, sections showed mature adipose tissue (*arrowed* in **a**–**c**) with multiple foci of loose cellular stoma entrap irregular sized vascular space, lined by single layer of epithelial cells, containing red cell with no evidence of nuclear pleiomorphism or malignancy seen (Hematoxylin and eosin stain; magnification ×40 and 100 respectively)

**Fig. 4:**
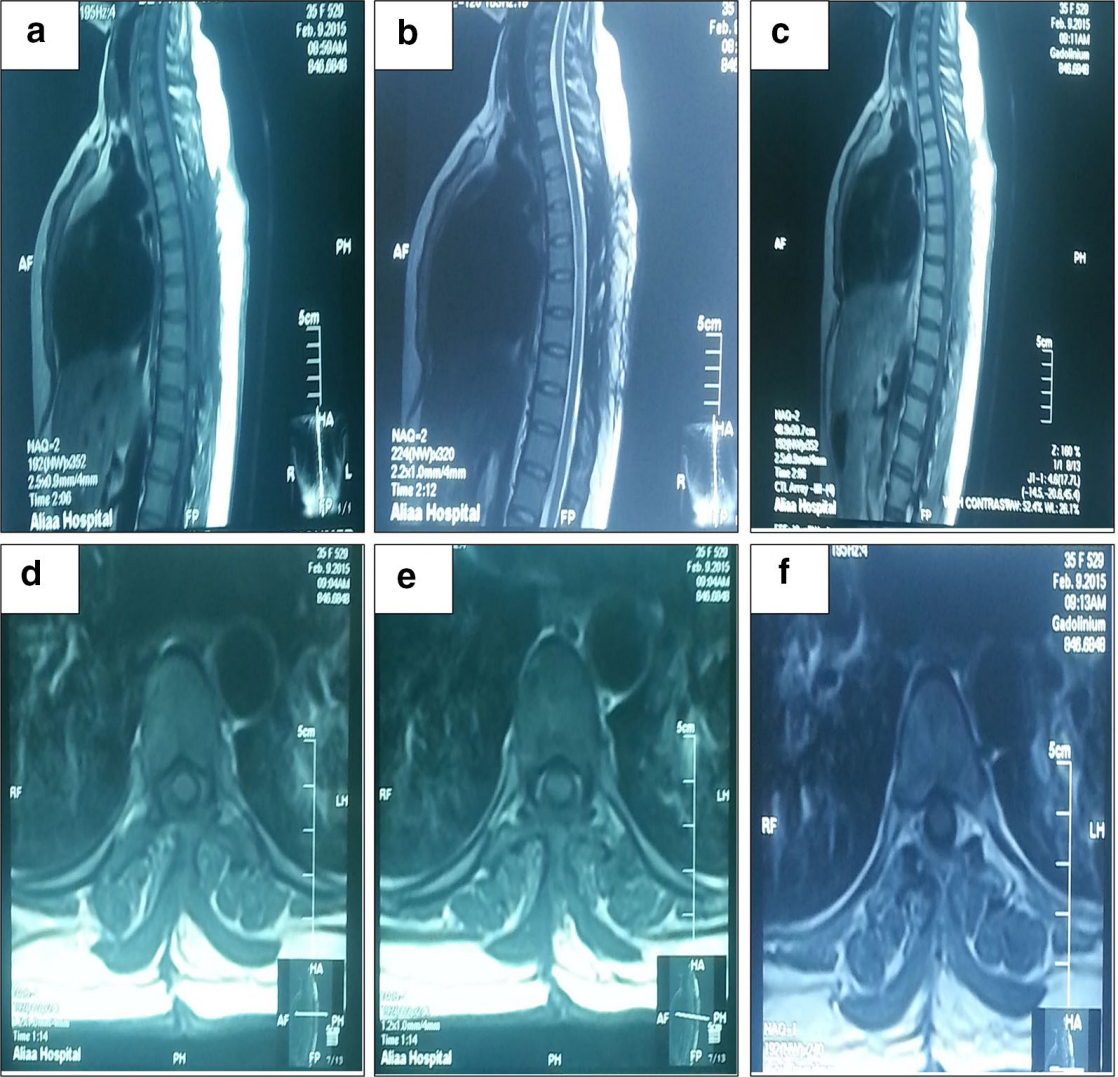
The scar of the surgery with no residual or recurrent extra-dural mass seen **a** T1 weighted sagittal MRI, **b** T2 weighted sagittal MRI, **c** T1 weighted sagittal MRI with contrast  all respectively showed no residuals or any recurrence of the lesion.cord is not compressed **d** Non contrast T1-weighted axial MRI **e** T2-weighted axial MRI and **f** post-contrast T1-weighted axial MRI showed total removal of the mass with marked areas of laminectomies and surgical scar

Patient was admitted for 5 days during which she showed no post-operative complications and no neurological deficit improvement, patient discharged on good condition, and sent for physiotherapy. Histopathology showed mature adipose tissue with multiple foci of loose cellular stroma entraps irregular sized vascular space features of which is favorable of Angiolipoma (Fig. 3). Three months later patient came for follow up and her condition showed improvement regarding her neurological motor and sensory functions (power improved to become grade 3+ and patient started to use walker), incontinence completely resolved. Post-operative follow up MRI was done (Fig. 4). On 9 months follow up patient showed regaining of her full power in both knees and ankles with PG 4+ in both hip joints with hyper-reflexia, mild hypertonia and equivocal response of planter reflex of both feet with recovery of her sensory functions.

On 1 year follow up patient showed regaining of her full power overall joints with mild hyperreflxia and no other neurological deficit.

## Discussion

Spinal angiolipomas are benign neoplasms with very rare occurrence in the spinal axis [2]. Angiolipomas of the spine represent about 0.14–1.2 % of all axial spinal tumors, 2–3 % of epidural spinal lesions, and 16–35 % of spinal lipomas [1]. Intracranial angiolipomas although rare but has been described.

There are only 142 cases with spinal extradural angiolipoma reported since 1890–2013 [3]. Liebscher [4] was the first to describe a case of spinal angiolipoma in 1901 which was diagnosed in autopsy. In 1960 establishment of the term angiolipoma was done by Howard and Helwig [5] as a separate histopathological category containing mature lipocytes and abnormally proliferated vessels. Lin [6] classified spinal epidural angiolipomas in the late 1974 into two main subtypes. The commonest type in which the spinal angiolipoma is encapsulated and non-infiltrating, and by surgical treatment it shows an excellent outcome and prognosis. The less common type is the infiltrating, non-encapsulated pathology, which showed an unfavourable outcome.

The histopathology of angiolipomas is poorly known and several theories have been placed in. Spinal angiolipomas may be classified as category in the middle between spinal lipomas and hemangiomas [7]. Once the tumor is being invasive with infiltrative features this would then represent a shift towards the hemangiomas as more likely possible diagnosis [8]. The main difference between spinal angiolipomas and lipomas is that the lipomas are commonly found in the lumbar and sacral areas and may be accompanied by spinal dysraphisim, while spinal angiolipomas are predominantly occur in the mid-thoracic area [9].

Pregnancy was an aggravating agent in the previously reported cases and pregnancy termination may lead to symptoms regression [10]. Drainage of the venous blood from the spine may be interfered with during pregnancy due to the massive compression on the abdominal and pelvic major veins which results in increasing epidural venous pressure which in turn increase the extracellular fluid volume, increasing abdominopelvic pressure in form of forceful valsalva maneuvers during vaginal delivery may suddenly aggravate symptoms and worsen condition [11]. Another suggested cause could be the vascular steal phenomenon which may lead to cord ischemia and compression which is exerted on the close cord areas due to the pulsations resultant from its high vascularity [12]. Hormonal factors and increased adipose tissues contents produced by pregnancy may also lead to enlargement of the extradural spine angiolipomas, also we can consider obesity as cause which may lead to symptoms exacerbation regardless the essential causative factor [8]. Hemorrhage or thrombosis within the tumor may cause abrupt deterioration in condition, two separate cases were mentioned in literature, one by Labram et al. [13] and the other by Anson et al. [14].

In gross specimen, the lesion is either to be covered by capsule or to be uncapsulated. Regarding the Histologic features, the components of the lesion is made up mainly of adipose tissue (mature type) and numerous vascular channels, the caliber of these channels is inconstant in range, ranging from nearly small capillary sized to large cavernous sized calibers. The fat tissues are of the mature lipocyte subtype and showed no significant abnormal findings. Lipocyte to vascular channels ratio is ranging from 1:3 to 2:3. Once the tumor has an abundant contents of smooth muscles they are sub-classified as angiomyolipomas (Labram et al. [12]).

Clinically, extradural spinal angiolipomas are similar to other spinal lesions especially those with benign nature. The presenting complains are commonly in form of loss of motor and sensory functions below the level of the affected part of the cord [15] which may lead to progressive weakness of the lower limbs and later on may be complicated by sphincter dysfunction [16].

MR Imaging is the modality of choice technique to diagnose all spinal extradural angiolipomas [11], showing hyperintenses signals on T1-weighted images without contrast which gives hint of their fatty content. In study conducted by Provenzale and Mclendon [11] it showed that the hypo-intense area noticed in the lesion on non-contrasted T1-weighted imaging study are indicative of their high vascularity whereas in T2-weighted images may be changeable, but are generally hyper-intense. Finally, most tumors enhance with gadolinium contrast administration [1]. Non-infiltrative type of spinal angiolipomas are most likely present at the posterior part of the extradural space with well demarcation lines from the surrounding tissues.

Total surgical removal of both infiltrating and non-infiltrating angiolipomas, is dependent on their location, which is in non-infiltrating type is mainly posterior and can be approached through posterior laminectomy approach, while in the infiltrating type it is likely to affect the vertebral body more than the posterior column, and it is best approached through either anterior, lateral or mixed approaches [3] and instrumentation of the influenced vertebral region is a favorable choice.

Total resection of the tumour prevent retrieval and grant improvement of the neurological deficits, one case has been reported as recurrence of an angiolipoma which was treated by successful surgery 12 years after the primary intervention [9] no other cases has been reported in both types of spinal angiolipomas even if complete removal could not be achieved [11]. Six deaths have been reported, from the nineteenth century up to date with no evidence or correlation to angiolipoma as the leading cause of death [6].

## Conclusions

Extradural spinal angiolipomas are known to be extremely infrequent benign neoplasms, it could be found along the whole spinal axis but it predominantly found in the mid-dorsal spine. The aetiology of angiolipomas is not well known but it may be arise from abnormal development and differentiation of pleuripotential mesenchymal cells which are part of the bilaminar embryological disc, which also is agreed to be mesenchymal hamartoma. Pregnancy is one of aggravating factors especially during vaginal delivery through interference of spinal venous blood flow and thus increase extradural venous pressure due to compression on the abdominal and pelvic major veins which is in turn exacerbate the symptoms.

Radiological features of angiolipoma on MRI show hyperintense lesions on T1WI, T2WI respectively, and almost all tumours will get the enhancement when gadolinium contrast is given.

Total surgical removal is the key of achieving excellent clinical outcome and prognosis without need for further adjuvant therapy.

## Consent

Written informed consent was obtained directly from the patient to allow publication of this case report and accompanying images.
